# The GraRS regulatory system controls *Staphylococcus aureus *susceptibility to antimicrobial host defenses

**DOI:** 10.1186/1471-2180-8-85

**Published:** 2008-06-02

**Authors:** Dirk Kraus, Silvia Herbert, Sascha A Kristian, Arya Khosravi, Victor Nizet, Friedrich Götz, Andreas Peschel

**Affiliations:** 1 Cellular and Molecular Microbiology Division, Institute for Medical Microbiology and Hygiene, University of Tübingen, Elfriede-Aulhorn-Str. 6, 72076 Tübingen, Germany; 2 Microbial Genetics, University of Tübingen, Tübingen, Germany; 3 Departments of Pediatrics, University of California, San Diego, La Jolla, California, USA; 4 Veterans Affairs San Diego Healthcare System, La Jolla, California, USA

## Abstract

**Background:**

Modification of teichoic acids with D-alanine by the products of the *dlt *operon protects Gram-positive bacteria against major antimicrobial host defense molecules such as defensins, cathelicidins, myeloperoxidase or phospholipase. The *gra*RS regulatory genes have recently been implicated in the control of D-alanylation in *Staphylococcus aureus*.

**Results:**

To determine the impact of the GraRS regulatory system on resistance to antimicrobial host defense mechanisms and virulence of *S. aureus*, we compared inactivation of *S. aureus *SA113 wild type and its isogenic *gra*RS deletion mutant by the human cathelicidin LL-37 or human neutrophil granulocytes *in vitro*, and the ability to cause infection *in vivo*. We show here that *gra*RS deletion considerably alters bacterial surface charge, increases susceptibility to killing by human neutrophils or the defense peptide LL-37, and attenuates virulence of *S. aureus *in a mouse infection model.

**Conclusion:**

Our results indicate that *S. aureus *can regulate its surface properties in order to overcome innate host defenses.

## Background

*Staphylococcus aureus*, a frequent cause of human infections, is highly resistant to antimicrobial factors of the innate immune system such as cationic antimicrobial peptides (CAMPs) [1,2] which are produced by epithelial cells and neutrophils [3,4]. These peptides generally contain 10–50 amino acids and have positive net charges [5]. Due to their cationic properties, CAMPs can easily bind to the highly negatively charged bacterial cell envelope and inactivate bacteria, e.g. by forming pores in the bacterial membrane leading to bacterial lysis [6]. *S. aureus *has evolved mechanisms to alter the anionic charge of cell surface components to gain resistance to a broad variety of cationic antimicrobial factors such as CAMPs [7], phospholipase A2 [8], myeloperoxidase [9], or lysozyme [10]. One such mechanism is based upon modification of phospholipids in the cytoplasmic membrane by introducing a positively charged lysyl group into anionic phosphatidylglycerol by the MprF protein, thereby neutralizing the net charge of the membrane surface [11,12]. A similar reaction is mediated by products of the *dlt*ABCD operon, which are responsible for attachment of positively charged D-alanine residues into negatively charged phosphate groups in the backbone of teichoic acids [7,9]. Mechanisms involved in the regulation of these resistance factors are not yet well understood in Gram-positive bacteria. Herbert *et al*. recently found that the *S. aureus *two-component regulatory system *gra*RS positively regulates expression of the *dlt *operon. In a *S. aureus *SA113 *gra*RS deletion mutant, the content of D-alanine in teichoic acids was reduced by 47% and the mutant showed reduced resistance to various antibiotics including polymyxin B, gallidermin, and vancomycin [10,13]. Accordingly, *gra*RS have previously been implicated in regulation of vancomycin intermediary resistance [14]. As the *dlt *operon plays a key role in *S. aureus *resistance to cationic antimicrobial host molecules, the *gra*RS system may be important in evasion of host defense mechanisms such as cationic antimicrobial peptides and neutrophil killing.

## Results

### The *gra*RS mutant shows altered cell surface charge but unaltered lysyl-phosphatidylglycerol (LPG) content

In order to study if reduced expression of the *dlt *operon upon *gra*RS disruption results in altered cell surface charge, we compared binding of the red-coloured, cationic protein cytochrome *c *to wild type *S. aureus *SA113 (WT), the isogenic *gra*RS mutant, and the plasmid-complemented mutant.

**Figure 1:**
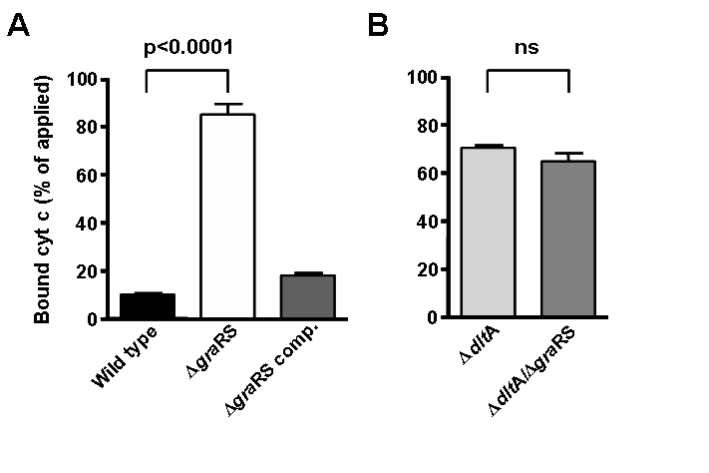
**Binding of cationic cytochrome *c *(cyt c) by *S. aureus *Sa113 wild type, *gra*RS mutant, and complemented *gra*RS mutant (A) and by the *dlt*A mutant and the *dlt*A/*gra*RS double mutant (B).** Bacteria were incubated with 500 μl cytochrome *c *solution (0.5 mg/ml) (A) or 750 μl cytochrome *c *solution (1 mg/ml) (B). The means and SEM of at least three independent experiments are shown. *P *values < 0.05 as calculated by Student's *t *test were regarded statistically significant.

The *gra*RS mutant bound significantly more cytochrome *c *than the WT or the complemented mutant (Fig. 1A), which is in accordance with the recently described reduced content of D-alanine residues in the teichoic acids of the mutant [10].

To analyse whether increased binding of cytochrome *c *by the *gra*RS mutant is in fact due to altered cell surface charge by decreased teichoic acid alanylation or the altered expression of other surface-exposed molecules, we also examined binding of cytochrome *c *to a *dlt*A deletion mutant, which completely lacks D-alanine substitution of teichoic acids [7], and a *dlt*A/*gra*RS double deletion mutant, which was generated by transducing the *gra*RS mutation into the *dlt*A mutant. Due to the high binding capacities of the *dlt*A and the *dlt*A/*gra*RS mutant we modified the conditions in order to prevent limitation of applied cytochrome *c*. Deletion of the *gra*RS genes in the *dlt *mutant background did not lead to further increased binding of cytochrome *c *compared to the *dlt*A mutant (Fig. 1B). This finding indicates that the increased binding of cytochrome *c *by the *gra*RS mutant is due to decreased alanylation of teichoic acids and that the resulting surface charge alteration does not result from other regulatory effects mediated by GraRS.

Increased binding of cationic proteins may also result from reduced *mpr*F expression and, accordingly, reduced lysylphosphatidylglycerol (LPG) content. To control for this possibility, we compared patterns of membrane lipids from log-phase bacteria by thin-layer chromatography. The amounts of LPG from WT and *gra*RS mutant were indistinguishable (data not shown), which corroborates recent findings that *mpr*F is not among the *gra*RS-regulated genes in *S. aureus *SA113 [10].

#### The *gra*RS mutant is more susceptible to killing by LL-37 and human neutrophil granulocytes *in vitro*

**Figure 2:**
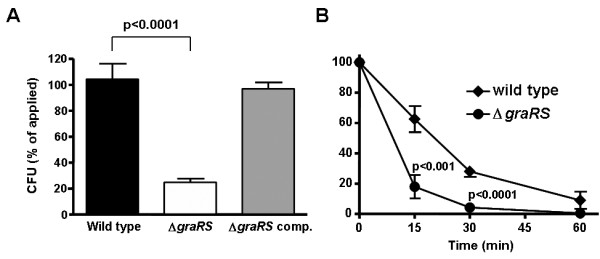
**(A) Inactivation of wild type (black bar), *gra*RS mutant (white bar) and complemented mutant (grey bar) by LL-37 after 20-min incubation with 20 μg/ml LL-37.** (B) Inactivation of wild-type (squares) and mutant bacteria (circles) by human neutrophils after 15, 30 and 60 minutes. The means and SEM of three independent experiments run in duplicate (A) and the means and SD of two independent experiments run in duplicate (B) are shown. *P *values < 0.05 as calculated by Student's *t *test were regarded statistically significant.

In an attempt to test whether the increased affinity of the *gra*RS mutant to cationic molecules leads to higher susceptibility to human host defense peptides, we compared inactivation of WT and *gra*RS mutant by the human cathelicidin LL-37. This antimicrobial peptide is active against *S. aureus *at high concentrations or long exposure [15] but in this experiment we chose conditions under which the LL-37 did not affect viability of the *S. aureus *WT. Accordingly, the WT and complemented mutant strains showed no significant decrease in CFU following LL-37 exposure, whereas the number of *gra*RS mutant bacteria recovered was only 25% of the original inoculum (Fig. 2A).

Next we investigated whether the *gra*RS mutant is killed faster than the parental strain by human neutrophils, which produce high amounts of LL-37 and other CAMPs as components of their antibacterial killing arsenal. The *gra*RS mutant was killed by neutrophils considerably faster than the WT strain. After 15 and 30 min, the recovered CFU of the *gra*RS mutant were significantly lower than those of the WT (Fig 2B). Of note, our previous studies had shown that altered alanylation of teichoic acids does not affect the rate of PMN phagocytosis [9]. Taken together, these data indicate that *gra*RS-mediated control of CAMP resistance mechanisms is of importance for *S. aureus *evasion from neutrophil killing.

#### Deletion of *gra*RS leads to attenuated virulence in a mouse infection model

To investigate the impact of reduced resistance of the *gra*RS mutant to neutrophil and CAMP-mediated killing on the ability of the bacteria to cause infections *in vivo*, we compared the virulence of WT and mutant bacteria in a mouse challenge model. Therefore female BALB/c mice (12 to 15 weeks old) were infected with *S. aureus *WT or *gra*RS mutant bacteria. 72 h after infection numbers of CFU/kidney were determined.

**Figure 3:**
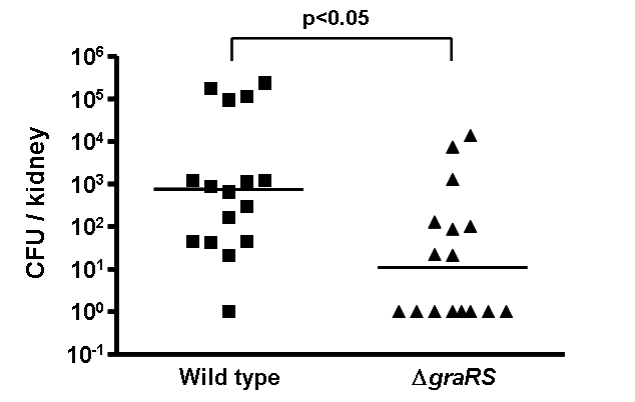
**CFU of wild-type and mutant bacteria in kidneys of mice 72 h after intraperitoneal infection with 1.2 × 10^8 ^bacteria.** For each strain 16 animals were tested. Medians are given as horizontal lines. A *P *value < 0.05 as calculated by Student's t test was regarded statistically significant.

Significantly less bacteria were detected in the kidneys of animals, which had been infected with the *gra*RS mutant than those infected with the WT bacteria. (Fig. 3) This finding is in coincidence with the increased susceptibility to clearance by CAMPs and neutrophils, corroborating the central importance of these host factors in innate defense.

## Discussion

A large variety of regulatory systems has been described in *S. aureus *during the last decades [16-18]. While many systems have been shown to control adhesion and toxin production [19], much less is known about the regulation of genes involved in the resistance to antimicrobial peptides, such as the *dlt *genes. However, the critical role of this operon in infection [7,9] suggests that it may be appropriately regulated in response to environmental stimuli. *Listeria monocytogenes *expresses the VirR transcription factor, which regulates both the *dlt *operon and the *mpr*F gene [20]. The *S. aureus *regulatory genes *rot *and *arl *have previously been shown to have a moderate influence on transcription of the *dlt *operon [21,22]. We have recently demonstrated that inactivation of the GraRS system leads to only 13% of the wild-type transcription level of the *dlt *operon and a decreased level of teichoic acid alanylation in *S. aureus *SA113 [10]. Other important genes regulated by *gra*RS in *S. aureus *include the *vra*FG genes involved in the resistance to vancomycin [10,13]. Similar data have recently been presented for the *Staphylococcus epidermidis aps *system [23]. This three component regulatory system is homologous to the *gra*RS system of *S. aureus*. However, when initially describing the *gra*RS system in *S. aureus*, Meehl et *al*. failed to recognize the three-component nature of this system [13]. Li et *al*. recently showed that deletion of *gra*S in the community-associated MRSA strain MW2 leads to decreased minimal inhibitory concentrations of some cationic antimicrobial peptides such as the human beta-defensin hBD3 or the human cathelicidin LL-37 and, similar to our studies, to decreased virulence of the mutant in a mouse infection model [24]. Furthermore, they could show that several important factors in CAMP resistance including the *dlt *operon and *mpr*F are activated by a diverse panel of antimicrobial peptides [24]. The fact that *mpr*F is not regulated by *gra*RS in *S. aureus *SA113 could be due to the deficiency of this strain in the global gene regulatory system *agr *[25] or to other genetic defects.

## Conclusion

We could show here that, besides its role in influencing the effectiveness of pharmacologic antimicrobials, the GraRS regulatory system plays a key role in resistance to natural antimicrobials of our innate immune system against *S. aureus *and merits further attention in an era of increasing reports of virulent and drug-resistant strains of this foremost human pathogen.

## Methods

### Strains and growth conditions

*Staphylococcus aureus *SA113 (WT), the isogenic *gra*RS deletion mutant [10], the plasmid-complemented *gra*RS deletion mutant [10], the isogenic *dlt*A deletion mutant [7] and the isogenic *dlt*A/*gra*RS double deletion mutant were inoculated in basic medium (BM; 1% tryptone, 0.5% yeast extract, 0.5% NaCl, 0.1% glucose, 0.1% K_2_HPO_4_) with aliquots of overnight cultures and incubated at 37°C until logarithmic phase was reached. In case of the complemented mutant, BM was modified by replacing glucose with 0.5% xylose to allow for expression of the plasmid-encoded *gra*RS genes [10].

The *gra*RS::*erm/dlt*A::*spc *double mutant was created by bacteriophage φ 11-mediated transduction of the *gra*RS*::erm *mutation into the *dlt*A*::spc *deletion mutant.

To prepare bacteria for the mouse infection model, precultures of the staphylococcal strains which were grown for 8 h in tryptic soy broth (TSB) were diluted 1:100 into fresh TSB and incubated for 18 h without shaking.

### Cytochrome *c *binding assay

Log-phase bacteria were harvested, washed twice with potassium phosphate buffer containing 0.01% human serum albumin (KPi buffer) and bacterial density was adjusted to an OD_600 _of 3. Bacteria from 1.5 ml aliquots were resuspended in 500 μl cytochrome *c *(Sigma) solution (0.5 mg/ml) in case of the wild type, the *gra*RS mutant and the complemented *gra*RS mutant and 750 μl cytochrome *c *(Sigma) solution (1 mg/ml) in case of the *dlt*A and the *dlt*A/*gra*RS double mutant, respectively, and incubated at 37°C. In order to prevent bacterial sedimentation, samples were vigorously shaken. After 15 min, samples were centrifuged and the supernatant was assayed photometrically at 410 nm.

### Lipid analyses

Comparison of membrane lipid patterns by extraction of polar lipids and subsequent thin-layer chromatography was performed as recently described [11].

### Inactivation assay with human defense peptide LL-37

Log-phase bacteria were harvested, washed twice with potassium phosphate buffer containing 0.01% human serum albumin (KPi buffer) and bacterial density was adjusted to an OD_600 _of 1.5. Samples (40 μl) with a final concentration of 20 μg/ml LL-37 were shaken at 37°C. After 20 min, 160 μl ice-cold KPi buffer was added to block further antimicrobial action and appropriate aliquots were plated on BM agar plates. After 24 h incubation at 37°C, CFU were enumerated.

### Inactivation assay with human neutrophil granulocytes

Bacteria were grown to logarithmic phase, washed, and adjusted in KPi buffer as described above. Neutrophils were isolated from peripheral blood of healthy volunteers by ficoll/histopaque gradient centrifugation as described previously [26] and resuspended in HBSS-HSA (HBSS containing 0.05% human serum albumin). Bacterial and neutrophil suspensions were mixed to final concentrations of 5 × 10^6^/ml bacteria and 2.5 × 10^6^/ml neutrophils. Bacteria were opsonized by addition of pooled human serum (Sigma) to a final concentration of 10%. Samples (500 μl) were shaken at 37°C. After 15, 30 and 60 min, aliquots were diluted in ice-cold water and vortexed vigorously to disrupt the neutrophils and halt bacterial killing. Appropriate dilutions were plated on BM agar plates and incubated at 37°C for 24 h for enumeration of CFU.

### Mouse infection model

All procedures involving animals were approved by the UCSD Animal Care Committee, which serves to ensure that all federal guidelines concerning animal experimentation are met.

Female BALB/c mice (12 to 15 weeks old) were infected intraperitoneally with *S. aureus *WT or the *gra*RS mutant. Briefly, precultures of the staphylococcal strains which were grown for 8 h in tryptic soy broth (TSB) were diluted 1:100 into fresh TSB, incubated for 18 h without shaking, washed twice in PBS, adjusted to 3 × 10^8 ^CFU/ml in PBS and 400 μl of these suspensions were injected intraperitoneally. 72 h after infection, mice were sacrificed, one kidney was aseptically removed, weighed, homogenized, and serially diluted in PBS for plating on Todd Hewitt agar plates. After 24 h incubation at 37°C, the numbers of CFU/kidney were determined.

## Authors' contributions

DK did all experiments except for the mouse infection model, which was done by SAK and AK. AP supervised research and wrote the paper. SH generated the *gra*RS/*dlt*A double mutant. AP, FG and VN conceived the study and analyzed results. All authors read and approved the final manuscript.
